# P-2203. High Rate of False Positives in Military Blood Donors Screening Positive for Hepatitis C Virus

**DOI:** 10.1093/ofid/ofae631.2357

**Published:** 2025-01-29

**Authors:** Rafaela Izurieta, Brian Casleton, Megan Phelps, Melita Gella, Glorimar Rivera, Theresa Casey, Angela Osuna, Ga O Jung, Erin Winkler, Heather Yun, Joseph Marcus, Samuel Owen

**Affiliations:** Brooke Army Medical Center, San Antonio, Texas; Armed Services Blood Bank San Antonio, San Antonio, Texas; DHA Lackland 59th MDW, San Antonio, Texas; Armed Services Blood Bank San Antonio, San Antonio, Texas; Armed Services Blood Bank San Antonio, San Antonio, Texas; BAMC, San Antonio, Texas; BAMC, San Antonio, Texas; 559th Medical Group, JBSA-Lackland, Texas; BAMC, San Antonio, Texas; Brooke Army Medical Center, San Antonio, Texas; Brooke Army Medical Center, San Antonio, Texas; Brooke Army Medical Center, San Antonio, Texas

## Abstract

**Background:**

While the overall rates of Hepatitis C virus (HCV) in military populations are low, positive post-donation screening represents the second most common indication for post-donation infectious deferrals in military blood donors. All donors who screen positive are permanently deferred from blood donation, regardless of follow-up testing in clinical laboratories. There is limited data on the follow-up evaluation of these blood donors. This study describes the confirmatory testing, access to appropriate subspecialty care, and treatment of blood donors who screened positive for HCV at a large military blood bank.

**Figure:**
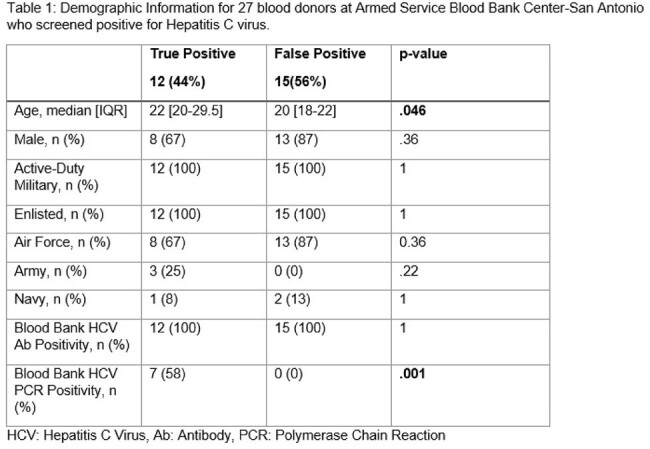

**Methods:**

All military blood donors between 2017-2023 at the Armed Service Blood Bank Center-San Antonio were screened for HCV by both antibody tests and nucleic acid tests, with positive antibody screening tests confirmed with an enzyme-linked immunosorbent assay (ELISA). Donors who screened positive had their medical charts reviewed to determine demographic information, follow-up evaluation, and treatment rates. On follow-up clinical diagnostic tests, patients with positive antibodies were determined to true positives (TP) and those with negative antibodies were determined to be FP.

**Figure:**
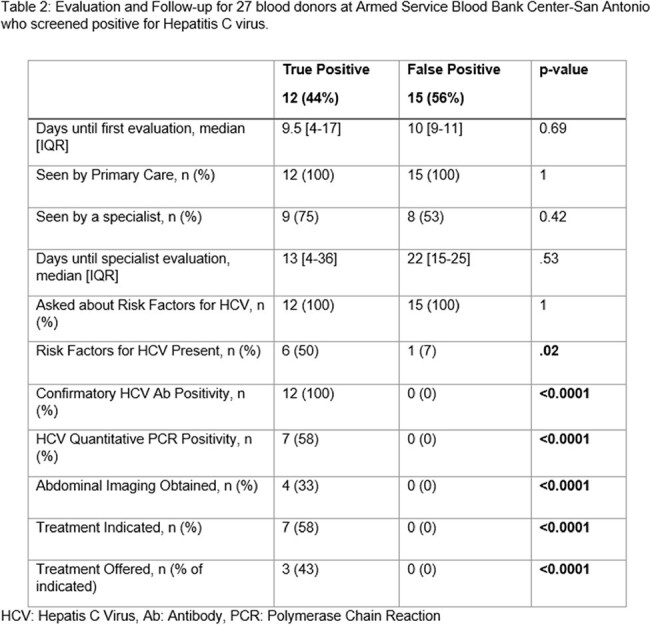

**Results:**

Of the 100,182 blood donors during the study period, 37 (0.04%) screened positive for HCV, of which follow-up information was available for 27 (73%). Of these 27, only 12 (44%) of donors were found to be TP. TP donors were more likely to be older (median 22 [20-29.5] vs. 20 [18-22], p=0.05) and have higher rates of positive nucleic acid testing (58% vs. 0%, p=0.001) after blood donation (Table 1). There was no difference in linkage to both primary and subspecialty care for TP and FP (Table 2). Of the TP, 7 (58%) were viremic, 3 (43%) of which received anti-viral therapy and 4 (57%) were administratively separated.

**Conclusion:**

While HCV is one of the most common infectious reasons for military service members who donate blood to be deferred from future donations, most positive screenings were FP. Future efforts should be made to re-enter these patients with FP screening tests back into the donor pool.

**Disclosures:**

All Authors: No reported disclosures

